# Locally advanced rectal cancer receiving total neoadjuvant therapy combined with nivolumab: a case report and literature review

**DOI:** 10.1186/s12957-022-02624-z

**Published:** 2022-05-26

**Authors:** Ryota Mori, Mamoru Uemura, Yuki Sekido, Tsuyoshi Hata, Takayuki Ogino, Hidekazu Takahashi, Norikatsu Miyoshi, Tsunekazu Mizushima, Yuichiro Doki, Hidetoshi Eguchi

**Affiliations:** Department of Gastroenterological Surgery, Graduate School of Medicine, Osaka University, 565-0871, 2-2, Yamadaoka, Suita, Osaka, Japan

**Keywords:** Locally advanced rectal cancer, Nivolumab, Total neoadjuvant therapy, Case report, Microsatellite instability, Chemoradiotherapy

## Abstract

**Background:**

The standard treatment for locally advanced rectal cancer (LARC) is preoperative chemoradiotherapy (CRT) followed by surgery and adjuvant chemotherapy. However, it has been suggested that intensification of neoadjuvant treatment with polychemotherapy in addition to CRT instead of as an adjuvant chemotherapy is better tolerated and associated with a higher pathological complete response (pCR) rate. This concept is known as total neoadjuvant therapy (TNT).

Recently, the addition of immunotherapy to preoperative CRT has been reported to be useful in LARC patients with mismatch-repair-deficiency and high levels of microsatellite instability (MSI-H), but there are no reports showing the therapeutic effect of nivolumab in combination with TNT.

**Case presentation:**

A 23-year-old man had frequent diarrhea. Preoperative examination revealed two adenocarcinomas in the rectum. His maternal grandmother had a rectal cancer patient who developed the disease at age 70s. The larger tumor was located at the peritoneal reflection, and its anterior border close to the prostate (<1 mm); there were eight enlarged pararectal lymph nodes. Considering the size and depth of the tumor, it was judged that radical resection with sufficient margins would be difficult. Therefore, it was decided that TNT would be performed. At first, CAPOX (capecitabine and L-OHP) was administered, followed by preoperative CRT (RT:50.4 Gy and capecitabine). During this period, genetic testing diagnosed this patient as MSI-H, so additional nivolumab was administered after CRT. Colonoscopy revealed that the larger tumor was no longer detectable, so robot-assisted intersphincteric resection and bilateral lateral lymph node dissection was performed. The diagnosis of pCR was made for the larger tumor and partial response was achieved for the smaller tumor, and no lymph node metastasis was found. Major complications were not observed and the patient was discharged on the 14th day after surgery. He was followed up without adjuvant chemotherapy and is alive and recurrence-free after 9 months.

**Conclusion:**

A case of LARC with MSI-H was treated with TNT with nivolumab, resulting in pCR and complete radical resection. This result suggests that nivolumab in addition to TNT can be an option as a preoperative strategy for LARC with MSI-H.

## Background

Colorectal cancers (CRC) are common worldwide, and rectal cancer accounts for one-third of them [1]. There have been remarkable advances in chemotherapy, radiation therapy, and surgical techniques with total mesorectal excision (TME), and combinations of these therapies have improved remarkably. These advancements have resulted in more patients with rectal cancer receiving anus-preserving curative surgery and lower local recurrence rates [2]. At present, the standard treatment for locally advanced rectal cancer (LARC) is preoperative chemoradiotherapy (CRT) followed by surgery with TME. In addition to this, adjuvant chemotherapy is considered for patients with stage II/III disease who have not received neoadjuvant chemotherapy. However, it has been suggested that intensification of neoadjuvant treatment with polychemotherapy, added before or after CRT instead of as an adjuvant chemotherapy, is better tolerated and associated with a higher pathological complete response (pCR) rate [2, 3]. Furthermore, treatment that can achieve a high rate of pCR is particularly useful for patients with low activities of daily living levels, because a wait-and-see option can be considered. This concept is known as total neoadjuvant therapy (TNT).

In recent years, immunotherapies have been playing an important role in cancer treatment. In particular, antibody drugs that block negative regulators of the immune system such as cytotoxic T lymphocyte antigen 4 (CTLA-4), programmed cell death 1 (PD-1), and programmed cell death 1 ligand 1 (PD-L1) have been shown to be effective in malignant melanoma and squamous cell carcinoma of the lung, and their efficacy has been verified in a variety of carcinomas [4–7]. In reports of the effects of PD-1 blockade in human tumors, only 1 of 33 patients with colorectal cancer had a response to this treatment [8]. This is a much smaller percentage than seen in melanomas, renal-cell cancers, and lung tumors, in which PD-1 blockade was found to be effective in most cases [8, 9]. Later it was revealed that patients who had a response to PD-1 blockade had colorectal cancers with high levels of microsatellite instability (MSI-H) [10]. In the Check Mate 142 study (NCT02060188), the PD1 inhibitor nivolumab was tested in patients with mismatch-repair-deficient (dMMR) or MSI-H metastatic CRC. Since this study showed a high response rate in terms of tumor progression, nivolumab received FDA regulatory approval in 2017 for the treatment of heavily mutated CRCs that have dMMR or MSI-H [11].

It has been known that RT has an effect on cancer cells because it disrupts various genes, including the ATM and p53 genes, causing cell cycle arrest and apoptosis. However, in recent years RT has also been shown to have an immunomodulating effect. RT upregulates tumor-associated antigen major histocompatibility complex (MHC), and as a result, it enhances antigen cross-presentation in the draining lymph node and increases T cell infiltration into tumors [12]. This fact implicates the ability of RT to promote an endogenous antigen-specific immune response and provides an additional mechanistic rationale for combining radiation with immunotherapy in the clinic [13]. In fact, in patients with locally advanced unresectable non–small cell lung cancer, the combination of conventional CRT and an anti–PD-L1 antibody yielded significant improvement in both progression-free survival (PFS) and overall survival (OS) [14, 15].

These reports suggest that the addition of immunotherapy to preoperative CRT may also be useful for LARC patients with dMMR and MSI-H. However, only one study has shown a benefit of preoperative CRT and nivolumab for LARC patients, and there are no reports showing the therapeutic effect of nivolumab in combination with TNT [16]. Therefore, we report a case of a patient with LARC with MSI-H who was treated with nivolumab in combination with TNT and achieved anus-preserving curative surgery.

## Case presentation

**Fig. 1:**
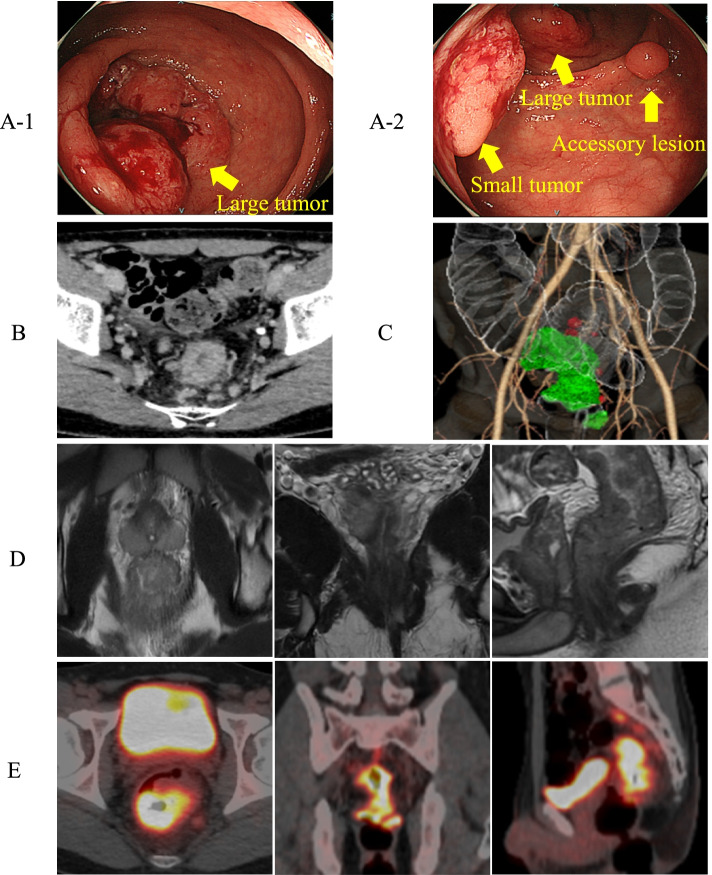
Pretreatment imaging findings. **A** CS images. Two tumors and an accessory lesion between them were identified in the rectum. The large main tumor was a circumferential advanced cancer and located on the second Houston valve (5 cm from anal verge), causing severe stenosis (**A1**). The small tumor was an early cancer 2 cm in diameter and it was about 0.5 cm from the dentate line (2.5 cm from the anal verge ). The accessory lesion was an adenoma 0.3 cm in diameter (**A2**). **B** Axial CT image. There was misty mesentery surrounding the large tumor and many enlarged pararectal lymph nodes. **C** CT colonography with three-dimensional reconstruction. **D** MRI images. The large tumor was located at the peritoneal reflection, with irregularity of the serous surface. The anterior border of the tumor was close to the prostate. **E** PET-CT images. ^18^F-FDG uptake was detected in tumors and lymph nodes. SUVmax: large tumor, 16.07; small tumor, 13.41

A 23-year-old man was admitted to a local doctor with symptoms of frequent diarrhea and was referred to our hospital due to mild anemia and positive fecal occult blood. He had no specific past illness but had a history of smoking in the past (Brinkman Index: 20 [10 × 2]). His maternal grandmother had a rectal cancer patient who developed the disease at age 70s. Initial laboratory data revealed a hemoglobin level of 12.0 and platelet count of 3.4 × 10^5^/μL. The tumor marker level of carcinoembryonic antigen (CEA) level was 2.0 ng/mL, and the carbohydrate antigen 19-9 (CA19-9) level was 19.3 ng/mL. There were no abnormal values in other blood test data. All blood test data are listed in Table 1. Colonoscopy (CS) identified two tumors in the rectum and an accessory lesion between them, and biopsies showed that the large main tumor was a poorly differentiated adenocarcinoma and the small tumor was well-differentiated adenocarcinoma (Fig. 4B). The large main tumor was a circumferential advanced cancer and located on the second Houston valve (5 cm from the anal verge), causing severe stenosis (Fig. 1A1). The small tumor was an early cancer 2 cm in diameter and it was about 0.5 cm away from the dentate line (2.5 cm from the anal verge) (Fig. 1A2). The accessory lesion was an adenoma 0.3 cm in diameter (Fig. 1A2). Computed tomography (CT) and magnetic resonance imaging (MRI) showed that the large tumor was located at the peritoneal reflection with irregularity of the serous surface and that the anterior border of the tumor was close to the prostate (<1 mm) (Fig. 1D). Misty mesentery surrounded the large tumor and there were eight enlarged pararectal lymph nodes (Fig. 1B, C). Positron emission tomography/computed tomography (PET-CT) revealed ^18^F-fluorodeoxyglucose (FDG) uptake in these tumors and lymph nodes but no distant metastases. The maximum standardized uptake (SUVmax) of ^18^F-FDG was 16.07 by the large and 13.41 by the small tumor (Fig. 1E). The clinical diagnosis was rectal cancer: cT4aN2bM0 cStageIIIc (large tumor) and cT1bN0M0 (small tumor) according to the Union for International Cancer Control Tumor, Node Metastasis Classification of Malignant Tumors, Eighth Edition [17].

**Table 1:** Preoperative blood test data are listed

Complete blood count	
WBC	4.8×10^3^/μL
Neu	51.1 %
RBC	4.4×10^6^/μL
Hb	12.0 g/dl
Ht	37.7%
Plt	3.4×10^5^/μL
**Tumor marker**	
CEA	2.0 ng/mL
CA19-9	19.3 U/mL
**Blood chemistry test**	
AST/GOP	14.0 U/L
ALT/GPT	10.0 U/L
CK	120 U/L
ALP	58.0 U/L
T-Bil	0.4 mg/dL
D-Bil	0.2 mg/dL
BUN	7.0 mg/dL
Cre	0.62 mg/dL
Na	141 mEq/L
K	4.1 mEq/L
Cl	103 mEq/L
TP	7.3 g/dL
Alb	4.0 g/dL
CRP	0.04 mg/dL

*WBC* white blood cell, *Neu* neutrophil, *RBC* red blood cell, *Hb* hemoglobin, *Ht* hematocrit, *Plt* platelet, *CEA* carcinoembryonic antigen, *CA19-9* carbohydrate antigen 19-9, *AST* aspartate aminotransferase, *ALT* alanine aminotransferase, *CK* creatinine phosphokinase, *ALP* alkaline phosphatase, *T-Bil* total bilirubin, *D-Bil* direct bilirubin, *BUN* blood urea nitrogen, *Cre* creatinine, *Na* natrium, *K* kalium, *Cl* chlorine, *TP* total protein, *Alb* albumin, *CRP* C-reactive protein

**Fig. 2:**
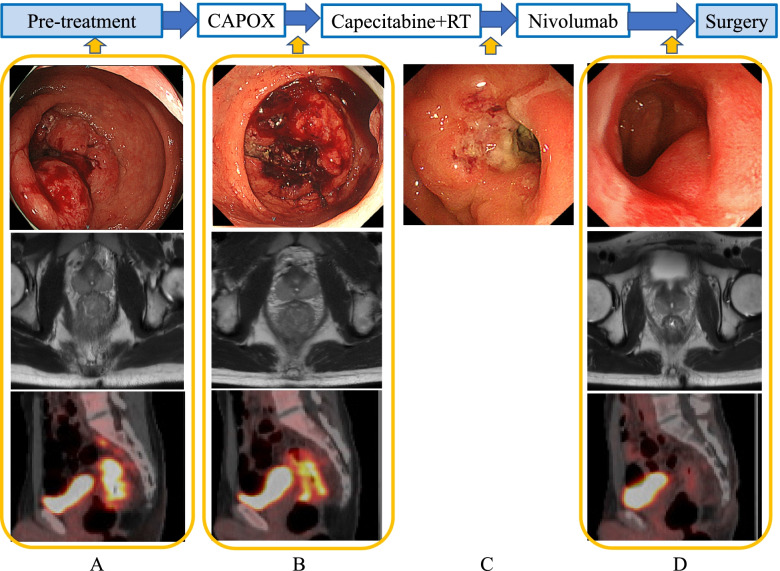
Clinical imaging in the course of treatment. After administration of CAPOX, the tumor did not shrink, but it started to shrink after capecitabine and radiotherapy. After the administration of nivolumab, the large tumor almost disappeared on imaging. **A** Colonoscopy, axial MRI, and sagittal PET images before treatment. **B** Colonoscopy, axial MRI, and sagittal PET images after CAPOX administration. **C** Colonoscopy images after capecitabine administration and radiotherapy. **D** Colonoscopy, axial MRI, and sagittal PET images after nivolumab administration

Considering the size and depth of the tumor, it was judged that radical resection with sufficient margins would be difficult. Therefore, the decision was made to perform TNT. At first, CAPOX (capecitabine 2000 mg/m^2^ for 14 days and L-OHP 130 mg/m^2^ on day 1) was administered every 3 weeks for 4 cycles. Imaging evaluation after CAPOX showed only slight tumor shrinkage (Fig. 2B). Next, preoperative CRT using capecitabine (1650 mg/m^2^ for 5 days per week) was performed. Radiotherapy was performed on the day of oral capecitabine, with a total dose of 50.4 Gy in 28 fractions. After CRT, partial tumor shrinkage was observed in CS (Fig. 2C). During this period, genetic testing diagnosed this patient as MSI-H, so additional nivolumab (240 mg/body every 2 weeks for 4 courses) was administered after CRT. This patient was under 50 years of age and had concurrent multiple cancers. So, although the patient did not meet the Amsterdam criteria II, he did meet the revised Bethesda Guidelines. Also considering the fact that this patient had MSI-H, Lynch syndrome was strongly suspected. Normally, the presence of BRAF V600E gene mutation and MMR gene mutation should be checked to make a definite diagnosis of Lynch syndrome, and the patient as well as his family members should be followed up. However, he did not have it tested because he and his family refused to have any further genetic testing done.

**Fig. 3:**
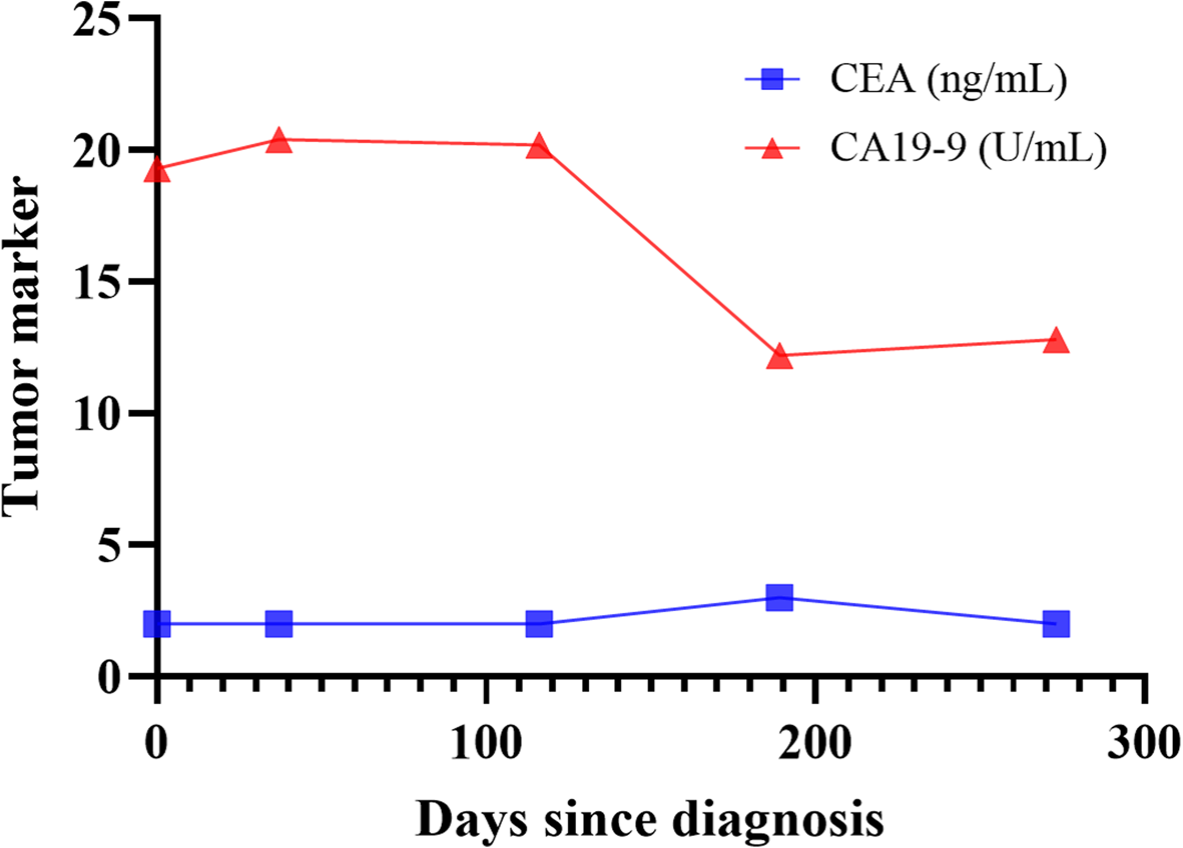
Changes in tumor markers over the course of treatment. Tumor markers were decreased after capecitabine administration and radiotherapy

During the course of preoperative treatment, no side effects were observed. CS revealed a mild stenosis in the area where the large tumor was located, but the viable tumor was no longer detectable (Fig. 2D). The small tumor was reduced in size but did not disappear. MRI showed residual rectal wall thickening, but the large tumor could not be detected and PET-CT showed no FDG accumulation in the tumor or lymph nodes (Fig. 2D). The tumor marker levels just before the surgery were CEA 2 ng/mL and CA 19-9 12.8 ng/mL, and CA19-9 was lower than before the treatment. As shown in Fig. 3, CA19-9 was decreased after the start of CRT.

**Fig. 4:**
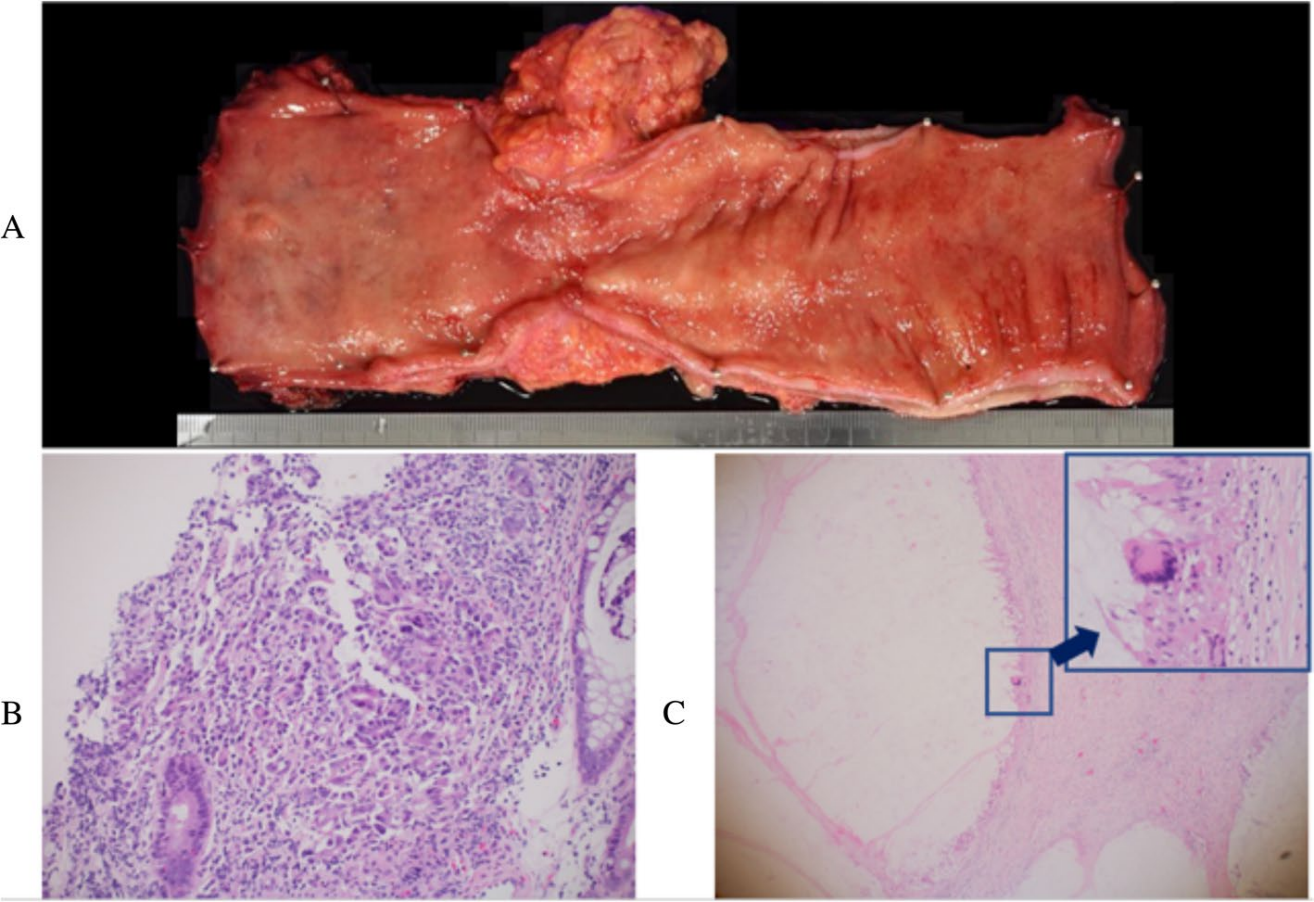
**A** Resected specimen of rectum. There was a mild stenosis in the area where the large tumor was located, but the tumor was no longer detectable. The small tumor was reduced in size, but did not disappear. **B** Pathological image of the large tumor before treatment. **C** Pathological image of the large tumor after treatment. There were cancer-free mucous nodules in the submucosa, intrinsic muscularis propria, and submucosa. The diagnosis of pCR was made

Robot-assisted intersphincteric resection and bilateral lateral lymph node dissection were performed. The operation time was 550 min, and the blood loss was 150 mL. There was no evidence of peritoneal dissemination or distant metastasis in the peritoneal cavity. Figure 4A showed the resected specimen. Pathological examination of the resected specimen was performed. In the area where the large tumor was located, there were cancer-free mucous nodules in the submucosa, intrinsic muscularis propria, and subserosa, and foreign body polynuclear giant cells and histiocytes were found around the nodules. The large tumor was therefore diagnosed as pCR (Fig. 4C). On the other hand, mucous nodules had spread to the submucosa in the area where the small tumor was located, but residual well-differentiated adenocarcinoma was found in the mucosal intrinsic layer (pTis). Consequently, the small tumor was diagnosed as having a partial response (pPR). There was no lymph node metastasis. Since no remaining cancer cells were found at the margins, it was diagnosed that complete resection was achieved. Major complications (Clavien–Dindo classification ≥IIIa) were not observed, and the patient was discharged on the 14th day after surgery. The patient was followed up without adjuvant chemotherapy and is alive and recurrence-free after 9 months.

## Discussion

LARC used to be treated only by surgery, but this was a problem because of the high local recurrence rate as well as the need for extensive surgery such as Miles operation and total pelvic exenteration. Based on the results of various studies, the standard treatment for LARC is preoperative CRT followed by surgery with TME, with adjuvant chemotherapy additionally considered [18–20]. However, adjuvant chemotherapy is not well tolerated, due to the effects of surgery. In the EORTC 22921 study, which is the largest adjuvant trial for LARC to date, adjuvant chemotherapy could be initiated in only 73% of patients and only 43% received 95% of the planned dose [21]. TNT, a combination of preoperative CRT and polychemotherapy, has therefore been receiving attention, and some studies have been reported.

TNT includes different strategies. As for chemotherapy, the combination of drugs, dosage, duration, and timing of administration have not been standardized. And as for CRT, the radiation dose, number of fractions, and time to surgery have also not been consistent. For these reasons, there is no consensus on whether the use of TNT is a better strategy or not, and how to perform TNT, but it has been reported to be better tolerated and associated with higher pCR rates in comparison with conventional treatment in a meta-analysis of these reports [2, 3]. Furthermore, in a meta-analysis of seven reports, patients who received TNT and surgery had better disease-free survival (DFS) (HR = 0.75, 95% CI 0.52–1.07, *P* = 0.1) and OS (HR = 0.73, 95% CI 0.59–0.9, *P* = 0.004) were favorable [2]. These results suggest that TNT not only improves pCR rates, but may also have a positive impact on prognosis.

Moreover, there is a discussion of whether induction chemotherapy and sequential CRT or CRT followed by consolidation chemotherapy is a better strategy. A randomized clinical trial (CAO/ARO/AIO-12) showed that CRT followed by consolidation chemotherapy had a higher pCR rate [22]. In addition, the preliminary result of OPRA (the Organ Preservation of Rectal Adenocarcinoma ) trial, a phase II randomized controlled trial, showed that there was no difference in 3-year DFS between the two groups. In this case, we performed TNT with induction chemotherapy and sequential CRT followed by nivolumab, but it is debatable whether CRT would have been better to do first or not.

In CRC, T cell infiltration into the tumor has long been reported to associate with a good prognosis, suggesting that the immune system regulates tumor progression [23]. The immune system distinguishes self from non-self through the binding of T cell receptors (TCR) on T cells to complexes of peptides with MHC class I molecules presented on the surface of all cells, including tumor cells [24]. However, recognition of peptide–MHC class I complexes by the TCR alone is insufficient for T cell activation [25]. TCR–MHC signaling pathways are modulated by co-stimulatory or co-inhibitory signals, by which tumor cells escape immune regulation [26]. Therefore, immunotherapies target co-inhibitory receptors, such as CTLA-4, PD-1, and PD-L1, on tumor cells or immune cells, in order to prevent T cell dysfunction and apoptosis and enhance T cells’ ability to kill tumor cells [25]. Nivolumab is a highly selective, fully humanized, IgG4 monoclonal antibody inhibitor of PD-1 and acts to selectively block receptor activation by PD-L1 and PD-L2 and thus release the immune response from PD-1 mediated inhibition [27]. Tumors with dMMR or MSI-H are likely to escape from immune regulation and are more likely to respond to immunotherapy. In this case, the patient had MSI-H and responded well to added nivolumab.

While immune therapy has improved the outcome of CRC patients, it has also resulted in the rise of unique immune-related adverse events (irAEs). These can be dermatologic, gastrointestinal/hepatic, endocrine, pulmonary, and cardiovascular. According to a recent review, the overall incidence of severe or life-threatening irAEs ranges from 10 to 15% for patients receiving anti-PD-1, and this toxicity is reportedly dose-independent [28]. In our case, there were no adverse effects of preoperative treatment, including immune-related complications.

The results of the VOLTAGE study, the first phase II trial of nivolumab in combination with preoperative chemoradiotherapy, have been reported [16]. According to this report, preoperative CRT followed by nivolumab and radical surgery was associated with only mild toxicity and increased the pCR rate, especially in patients with MSI-H (60 %) [16]. In our case, the course of treatment response suggested that TNT with nivolumab improved the outcome of patients with MSI-H for the first time. Since this is a report of a single case, future clinical trials are awaited.

## Conclusion

We report a case of LARC with MSI-H that was treated with TNT with nivolumab, resulting in pCR and complete radical resection without any side effects. This result suggests that nivolumab in addition to TNT can be an option as a preoperative strategy for LARC with MSI-H.

## Data Availability

These datasets generated and/or analyzed during the current study are publicly available from the corresponding author on reasonable request.

## Abbreviations

LARC: Locally advanced rectal cancer
CRT: Chemoradiotherapy
pCR: Pathological complete response
TNT: Total neoadjuvant therapy
dMMR: Mismatch-repair-deficiency
MSI-H: High levels of microsatellite instability
MHC: Major histocompatibility complex
CS: Colonoscopy
CRC: Colorectal cancers
TME: Total mesorectal excision
CTLA-4: Cytotoxic T lymphocyte antigen 4
PD-1: Programmed cell death 1
PD-L1: Programmed cell death 1 ligand 1
PFS: Progression-free survival
DFS: Disease-free survival
OS: Overall survival
CEA: Carcinoembryonic antigen
CA19-9: Carbohydrate antigen 19-9
CT: Computed tomography
MRI: Magnetic resonance imaging
FDG-PET: Positron emission tomography/computed tomography
FDG: Fluorodeoxyglucose
SUVmax: Maximum standardized uptake
irAEs: Fimmune-related adverse events
WBC: White blood cell
Neu: Neutrophil
RBC: Red blood cell
Hb: Hemoglobin
Ht: Hematocrit
Plt: Platelet
AST: Aspartate aminotransferase
ALT: Alanine aminotransferase
CK: Creatinine phosphokinase
ALP: Alkaline phosphatase
T-Bil: Total bilirubin
D-Bil: Direct bilirubin
BUN: Blood urea nitrogen
Cre: Creatinine
Na: Natrium
K: Kalium
Cl: Chlorine
TP: Total protein
Alb: Albumin
CRP: C-reactive protein
